# Recommendations from the Italian Society of Pediatric Orthopaedics and Traumatology for the management of pediatric orthopaedic patients during the COVID19 pandemic and post-pandemic period in Italy

**DOI:** 10.1186/s13052-020-00911-7

**Published:** 2020-10-08

**Authors:** Giovanni Trisolino, Carlo Enrico Origo, Nando De Sanctis, Daniela Dibello, Pasquale Farsetti, Cosimo Gigante, Pasquale Guida, Lorenza Marengo, Elena Panuccio, Renato Maria Toniolo, Fabio Verdoni, Antonio Memeo

**Affiliations:** 1 Unit of Pediatric Orthopaedics and Traumatology, IRCCS Istituto Ortopedico Rizzoli, Via Pupilli 1, 40136 Bologna, Italy; 2 Orthopaedics and Traumatology Unit, Cesare Arrigo Children’s Hospital, Alessandria, Italy; 3 Unit of Pediatric Orthopaedics and Traumatology, Campolongo Hospital, Marina di, Eboli, SA Italy; 4 Unit of Pediatric Orthopaedics and Traumatology Giovanni XXIII Children’s Hospital, University of Bari, Bari, Italy; 5 Department of Orthopaedics Surgery, University of Rome “Tor Vergata”, Rome, Italy; 6 Department of Woman and Child Health, Pediatric Orthopaedic Unit, Padua General Hospital, Padua, Italy; 7 Unit of Pediatric Orthopaedics and Traumatology, Azienda Ospedaliera di Rilievo Nazionale Santobono Pausillipon, Naples, Italy; 8 Unit of Pediatric Orthopaedics and Traumatology, I.R.C.C.S. Istituto Giannina Gaslini, Genoa, Italy; 9 Department of Paediatric Orthopaedics and Traumatology, Centro Specialistico Ortopedico Traumatologico Gaetano Pini-CTO, P.zza A. Ferrari 1, 20122 Milan, Italy; 10 Unit of Pediatric Orthopaedics and Traumatology, IRCCS Bambino Gesù Paediatric Hospital, Rome, Italy; 11 Unit of Pediatric Orthopaedics and Traumatology, IRCCS Istituto Galeazzi, Milan, Italy

**Keywords:** COVID-19, SARS-Cov-2, Pediatric Orthopaedics, Recommendations

## Abstract

The rapid spread of the COVID-19 outbreak in Italy has dramatically impacted the National Healthcare System, causing the sudden congestion of hospitals, especially in Northern Italy, thus imposing drastic restriction of almost all routine medical care. This exceptional adaptation of the Italian National Healthcare System has also been felt by non-frontline settings such as Pediatric Orthopaedic Units, where the limitation or temporary suspension of most routine care activities met with a need to maintain continuity of care and avoid secondary issues due to the delay or suspension of the routine clinical practice. The Italian Society of Pediatric Orthopaedics and Traumatology formulated general and specific recommendations to face the COVID-19 outbreak, aiming to provide essential care for children needing orthopaedic treatments during the pandemic and early post-peak period, ensure safety of children, caregivers and healthcare providers and limit the spread of contagion.

## Introduction

The Coronavirus Disease 2019 (COVID-19) caused by severe acute respiratory syndrome coronavirus 2 (SARS-CoV-2) was first identified in Wuhan (Hubei province, China) in late 2019, rapidly spread worldwide, and, on March 11th, 2020, the World Health Organization (WHO) officially declared COVID-19 as a pandemic [1]. In the same days, the government of Italy imposed a national quarantine that locked down for about 2 months all commercial and industrial activities (with some exceptions), school and universities, sports, cultural and leisure activities [2]. As the contagion rate and death toll COVID-19 related continued to decrease, on April 26th, 2020 the Prime Minister announced the so-called “phase 2” for the reopening and resumption of the activities scheduled for May 18th, 2020. On May 18th, 2020, the WHO reported 4,628,903 confirmed cases of COVID-19 (225,435 cases in Italy), including 312,009 deaths (31,908 in Italy) [1]. So far, along with the Hubei province, Italy (and in particular Northern Italy) has had the longest period of lockdown during the COVID-19 pandemic (almost 3 months in Northern Italy).

The rapid spread of the COVID-19 outbreak has immediately caused the congestion of many hospitals, thus imposing the temporary interruption of all non-essential medical cares. This exceptional adaptation of the Italian National Health Service was significantly evident also within non-frontline healthcare settings such as Pediatric Orthopaedic Units, where limitation and temporary suspension of most routine care activities was necessary to reduce the risk of infection in patients, families, and healthcare providers and to reallocate healthcare personnel from routine tasks to emergency.

On the other side, the limitation of healthcare services to the essential ones, along with the general reluctance among people to access care for fear of COVID-19 exposure, led to a in increased risk and worsening of COVID19-unrelated diseases. In Italy, an increase of death and worsening pediatric diseases due to delayed access or provision of care has been reported [3]. Therefore, a need for preventing the risk of delays in access to care is essential especially in children, in order to avoid complications due to the alteration or suspension of the typical patient care.

**Fig. 1:**
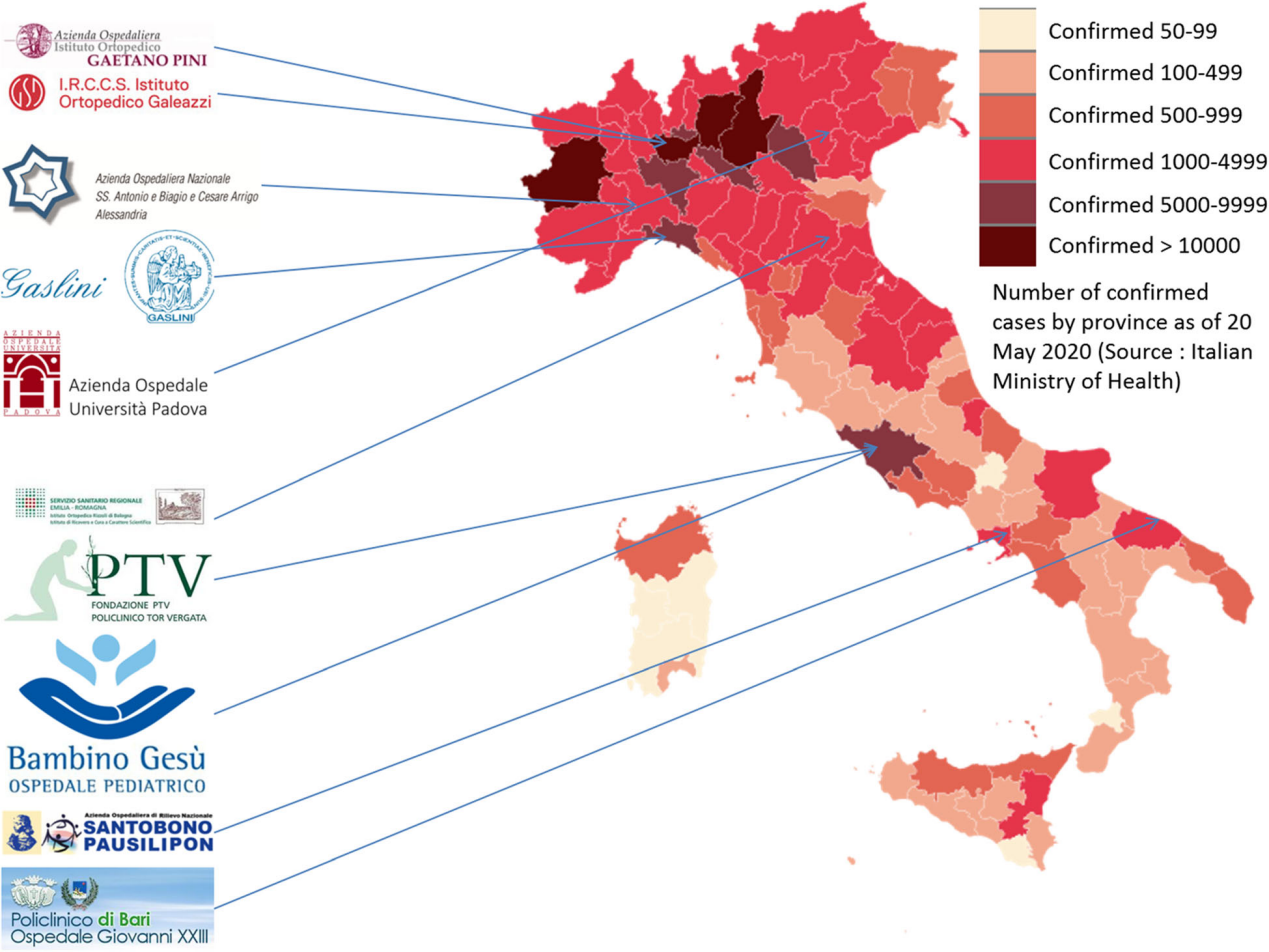
Location of the pediatric hospitals involved in the present study in relation to the COVID-19 epidemic density in the Italian province (Source: Italian Ministry of Health. Last update: May, 20th 2020)

Given the profound uncertainty about the actions to be taken, on March 2020, the Advisory Board of the Italian Society of Pediatric Orthopaedics and Traumatology (SITOP) launched an initiative to gather local experiences and epidemic risk management protocols from nine tertiary referral centers for Pediatric Orthopaedics and Traumatology in Italy concerning the COVID-19 outbreak (Fig. 1).

This produced an initial emergency document that was approved on April 9th, 2020, concerning the measures to be adopted during the pandemic period and a second document that was approved on May 18th, 2020 and sent to the Italian Society of Orthopaedics and Traumatology to be included in a national panel of recommendations for orthopaedic surgeons.

## Aims

The present document provides general and specific recommendations for pediatric orthopaedic surgeons, who face the so-called “pandemic period” and “post-peak period”. Additional protocols and guidelines concerning the management of pre-pandemic and inter-pandemic periods are included into the WHO Guidance Document [4].

The recommendations listed in this document have the following aims:

1. provide essential care to pediatric patients needing orthopaedic treatments during the COVID-19 pandemic and early post-peak period;
2. ensure safety of children and caregivers in case of hospital admission;
3. ensure safety of medical staffs;
4. limit the spread of outbreak.

These recommendations should be considered not as mandatory guidelines but rather as a support to reassure families and pediatric orthopaedic surgeons regarding the possibility to treat children safely during the pandemic and post-peak period. Therefore, they can be adapted and modified depending on locally available resources and personal experience of the pediatric orthopaedic staff.

We believe that they could be useful for future management of upcoming pandemics or during an eventual second wave of COVID-19, after that safety and effectiveness of these approaches will be analysed, using the data collected during this period. We are also confident that the experience gained across some of the main Italian pediatric orthopaedic hospitals could be helpful also in other countries which are facing the pandemic at an earlier phase.

## Pandemic period

### General recommendations

Separate access to triage areas, emergency, wards, outpatient clinics, operating theatres must be defined, based on suspected or confirmed infection (path A: SARS-COV2 negative patients. Path B: SARS-COV2 positive or suspected patients). The paths must be physically separated, limiting asmuch as possible the possibility of communication between path A and path B [5]. Staff within path B must take Level III precautions and protective measures (see Table 1). The personnel within the path A and B should take precautions and protection measures, according to the current WHO guidelines [6] (Table 1). Filter areas and COVID-19 testing using nasopharyngeal swabs must be implemented for patients/caregivers scheduled for urgent/elective surgery before admission to hospital.

**Table 1:** Sars-CoV-2 related personal protection management. Adapted from the WHO guidance for rational use of personal protective equipment for COVID-19 [6]. FFP2/3 = Filtering Facepiece Particles. ^a^The screening procedure refers to prompt identification of patients with signs and symptoms of COVID-19. ^b^ AGP: tracheal intubation, non-invasive ventilation, tracheotomy, cardiopulmonary resuscitation, manual ventilation before intubation, bronchoscopy.^c^: This category includes the use of no-touch thermometers, thermal imaging cameras, and limited observation and questioning, all while maintaining a spatial distance of at least 1 m. ^d^: the number of visitors should be restricted. If visitors must enter a COVID-19 patient’s room, they should be provided with clear instructions about how to put on and remove PPE and about performing hand hygiene before putting on and after removing PPE; this should be supervised by a health care worker

Setting (healthcare facilities)	Target (personnel or patients)	Activity	Type of PPE or procedure
**Inpatient facilities**			
**Screening**^**a**^ **Clinical triage for prioritization of care according to severity should be performed in separate area for individuals with symptoms and signs**	Health care workers	Preliminary screening not involving direct contact^c^	• Maintain physical distance of at least 1 m. • Ideally, build glass/plastic screens to create a barrier between health care workers and patients. • No PPE required. • When physical distance is not feasible and yet no patient contact, use mask and eye protection.
	Patients with symptoms suggestive of COVID-19	Any	• Maintain physical distance of at least 1 m. • Provide medical mask if tolerated by patient. • Immediately move the patient to an isolation room or separate area away from others; if this is not feasible, ensure spatial distance of at least 1 m from other patients. • Perform hand hygiene and have the patient perform hand hygiene.
	Patients without symptoms suggestive of COVID-19	Any	• No PPE required. • Perform hand hygiene and have the patient perform hand hygiene.
**Patient room/ward**	Health care workers	Providing direct care to COVID19 patients, **in the absence** of aerosol generating procedures	• Medical mask. • Gown. • Gloves. • Eye protection (goggles or face shield). • Perform hand hygiene.
	Health care workers	Providing direct care to COVID19 patients in settings where aerosol generating procedures are frequently in place^b^	• Respirator N95 or FFP2 or FFP3 standard, or equivalent. • Gown. • Gloves. • Eye protection. • Apron. • Perform hand hygiene.
	Cleaners	Entering the room of COVID-19 patients	• Maintain physical distance of at least 1 m. • Medical mask. • Gown. • Gloves. • Perform hand hygiene.
	Visitors^d^	Entering the room of COVID-19 patients	• Maintain physical distance of at least 1 m. • Medical mask. • Gown. • Gloves. • Perform hand hygiene
**Areas of transit where patients are not allowed (e.g. cafeteria, corridors)**	All staff, including healthcare workers.	Any activity that does not involve contact with COVID-19 patients	• Maintain physical distance of at least 1 m. • No PPE required. • Perform hand hygiene
**Laboratory**	Lab technician	Manipulation of respiratory samples. Specimen handling for molecular testing would require BSL-2 or equivalent facilities. Handling and processing of specimens from cases with suspected or confirmed COVID19 infection that are intended for additional laboratory tests, such as haematology or blood gas analysis, should apply standard precautions.	• Maintain physical distance of at least 1 m. • Medical mask. • Eye protection. • Gown. • Gloves. • Perform hand hygiene.
**Administrative areas**	All staff, including health care workers.	Administrative tasks that do not involve contact with COVID-19 patients.	• Maintain physical distance of at least 1 m. No PPE required. • Perform hand hygiene.
**Outpatient facilities**			
**Screening**^**a**^**/triage**	Health care workers	Preliminary screening not involving direct contact^c^	• Maintain physical distance of at least 1 m. • Ideally, build glass/plastic screens to create a barrier between health care workers and patients. • No PPE required. • When physical distance is not feasible and yet no patient contact, use mask and eye protection. • Perform hand hygiene
	Patients with symptoms suggestive of COVID-19	Any	• Maintain spatial distance of at least 1 m. • Provide medical mask if tolerated. • Perform hand hygiene.
	Patients without symptoms suggestive of COVID-19	Any	• No PPE required. • Perform hand hygiene
**Waiting room**	Patients with symptoms suggestive of COVID-19	Any	• Provide medical mask if tolerated. • Immediately move the patient to an isolation room or separate area away from others; if this is not feasible, ensure spatial distance of at least 1 m from other patients. • Have the patient perform hand hygiene.
	Patients without respiratory symptoms	Any	• No PPE required. • Perform hand hygiene.
**Consultation room**	Healthcare workers	Physical examination of patient with symptoms suggestive of COVID-19	• Medical mask. • Gown. • Gloves. • Eye protection. • Perform hand hygiene.
	Healthcare workers	Physical examination of patients without symptoms suggestive of COVID-19	• PPE according to standard precautions and risk assessment. • Perform hand hygiene.
	Patients with symptoms suggestive of COVID-19	Any	• Provide medical mask if tolerated. • Hand hygiene and respiratory etiquette
	Patients without symptoms suggestive of COVID-19	Any	• No PPE required. • Have the patient perform hand hygiene.
	Cleaners	After and between consultationswith patients with respiratory symptoms	• Medical mask. • Gown. • Heavy-duty gloves. • Eye protection (if risk of splash from organic material or chemicals). • Closed work shoes. • Perform hand hygiene
**Administrative areas**	All staff, including healthcare workers	Administrative tasks	• Maintain physical distance of at least 1 m • No PPE required. • Perform hand hygiene.

### Outpatient care

Cancel all deferrable outpatient appointments with clear notice to the patient through their phone numbers, in order to reduce the circulation of users and staff within hospital structures. Regular follow-up visits should be temporarily suspended.

Appointments should be preserved for children requiring non postponable post-operative care (for example percutaneous K-wires removal, cast removal or renewal, medication of complicated wounds).

Children with recent onset and progressive exacerbation of pain or functional impairment, even in absence of trauma, should be also visited in person, in order to rule out severe diseases such as bone tumors, infections and acute rheumatic diseases (septic arthritis, osteomyelitis, juvenile arthritis) or severe developmental orthopaedic diseases (SCFE, Perthes disease, etc..), which may necessitate non deferrable treatments.

Newborns should be visited in person in agreement with neonatologist or pediatric recommendation. The interval between appointments must be prolonged, in order to avoid crowding the waiting rooms and allow disinfection of the outpatient facilities. In any case, telemedicine must be encouraged whenever possible. Follow up imaging should be taken near the locality of the patient and e-mailed to the institute, only if likely to make a significant change to care [7, 8].

### Emergency care

Carefully define and organize the management and priority of urgent and emergent interventions. Children should be visited and treated by surgeons experienced in pediatric orthopaedics and nonoperative treatments should be encouraged and performed directly by the experienced senior surgeons. Whenever feasible, use and teach parents to remove self-removable casts or splints, to reduce the follow-up requirement. When considering surgical management, the following priority protocol should be adopted:

1. Emergent surgical intervention (< 24 h): open fractures or acute injuries with severe neurovascular involvement and potential risk for life, limb loss, or permanent damage. Screen the patient using rapid response SARS-COV 2 tests (nasopharyngeal swab). If absent, or if surgery is required in less than 4–6 h, treat the patient as COVID-19-positive, until proven otherwise, in order to minimize infection spread. Provide patient with FFP1 during transit from emergency to operation theatre. Clean her/his hands with antiseptic solutions. Protect the surgical staff.
2. Urgent surgical intervention (24–72 h): displaced unreducible and/or unstable acute closed fractures and dislocations, soft tissue injuries without severe neurovascular damage. If operative treatment is required, screen the patient using rapid response SARS-COV 2 test (nasopharyngeal swab). Patient must stay in the filter area until the test response. If the child is COVID-19-negative, he/she should be managed within the path A. If the child is COVID-19-positive he/she should be managed within the path B. Surgery must be performed by expert pediatric orthopaedic surgeons, maximizing the use of closed reduction, percutaneous pinning, resorbable sutures and self-removable casts of splints. Inpatient care should be minimized as much as possible.

### Surgical care

In case of elective surgery, a strict priority must be maintained in the waiting list, especially if the duration of the pandemic period cannot be precisely estimated [7, 8]. The Advisory Board of the SITOP has provided a panel of priority levels in order to safely schedule deferrable surgical treatments, reducing the risk of missing children who require non postponable operations, during the pandemic and post peak period (see Table 2).

**Table 2:** priority class for elective pediatric orthopedic surgery

Priority	A	B	C	D
**Type of surgery**	• Surgery for malignant or aggressive bone and soft tissue tumors. • Biopsies for suspected malignancies. • Septic arthritis requiring arthroscopic lavage/sampling/evacuation. • Slipped capital femoral epiphysis. • Misdiagnosed, neglected fractures or fractures displaced at follow-up. • Hardware-related complications (infection, migration…). • Nerve injuries or compression with recent onset palsy not responding to nonoperative treatments. • Locked knee, bucket handle meniscal tear, loose bodies, OCD fragments.	• Staple or guided growth hardware removal in case of overcorrection. • Ponseti method for CTEV in older newborns (3–6 months). • Closed/open reduction and cast for CDH in older newborns (3–6 months).	• Minimally invasive surgery (percutaneous tenotomies, subtalar arthroereisis). • Arthroscopic procedures. • Procedures that should be done at a definite range of age (for example epiphysiodesis and hemiepiphysiodesis at transitional age, treatments for congenital knee or foot and ankle dislocation, before start walking).	• Surgical treatments in skeletally mature children. • Limb lengthening procedures. • Osteotomies of pelvis and long bones. • Arthrodesis. • Spinal surgery for scoliosis.

This priority panel has considered several factors such as:

1. duration of the pandemic period and the local epidemic density;
2. availability and accessibility of hospitals and surgical rooms;
3. characteristics and severity of the pediatric orthopaedic disease;
4. range of age of patients, since the favourable outcomes and even the feasibility of some pediatric orthopaedic procedures (for example, closed or open reduction of severe CDH, Ponseti method for severe CTEV, growth modulation procedures,) are significantly impacted by age at treatment;
5. type of operation and surgical technique, since some procedures are at higher risk for dissemination of the infection.

Based on this priority panel elective surgical procedures should be categorized in four classes:

1. Priority A: intervention that should be performed within 30 days from the start of the pandemic phase;
2. Priority B intervention that could be performed within 3 months from the start of the pandemic phase. Cases belonging to this priority should carefully monitored both for age of the child and local epidemic density, in order to start treatments as much as possible close to the start of the post-peak phase
3. Priority C: intervention that could be performed within the first 6 months from the start of the post-peak period
4. Priority D: intervention that can be safely performed at the end of the pandemic.

Healthcare providers must inform the family that only one parent/caregiver can assist the child during hospitalization. At hospital admission, both child and parent/caregiver must be mandatory screened for SARS-COV2. In patient care must be organized so that only one child and only one parent/caregiver per room are allowed. If the patient or parents are known or suspected to be COVID 19-positive, the operation should be postponed until the COVID tests are negative. As tests can occasionally become negative after more than 1 month, if such delay can seriously threaten the health of the child due to the orthopaedic pathology, the surgeon must evaluate if the treatment must be anticipated and the patient can be safely managed through the path B.

Surgical treatments belonging to the priority class A or B should not be suspended during the pandemic period especially if the duration of this period lasts for more than two to 3 months. The possibility to continue with high priority interventions in children should be preserved. Wherever possible, depending on the local setting of healthcare services, the management of such pediatric orthopaedic conditions should be centralized in non-COVID hospitals.

## Post-peak period

Strict surveillance of the infection should be maintained and all measures taken to ensure patient safety in the hospital (filter areas, separated paths, screening measures, protections, distancing measures) should be continued in anticipation of a possible second wave of infection. Outpatient care can be progressively resumed, maintaining the distancing measures. Wherever feasible, local health facilities should coordinate in order to redistribute outpatient visits, avoiding overcrowding of hospital facilities. Patients should be contacted by phone and/or e-mail to confirm re-appointment.

Emergency and surgical care should maintain the same organization, recommendations and priority classes of the pandemic period. Elective surgery can be gradually resumed, respecting the order of priority established during the pandemic phase. Priority B-C surgical treatments could be prioritized close to the end of the pandemic period, depending on the local stage of outbreak, the accessibility and availability of operating rooms.

When elective surgery must be scheduled in the immediate post-peak period consider to prioritize those operations, which:

1. require minimally invasive, arthroscopic or percutaneous techniques;
2. do not require post-operative intensive care unit recovery;
3. can be safely managed within day-surgery or with minimal in-patient care (possibly < 3 days);
4. when possible, postponing at late stage of post-peak patients with comorbidities (especially cardiovascular or respiratory);
5. must be performed within a definite range of age (for example guided growth procedures).

Wherever possible we recommend using surgical techniques that significantly reduce the risk of aerosol-generating procedures (see Table 3). Local/regional anesthesia should be preferred to invasive airway management whenever possible for elective orthopaedic procedures of the upper and lower extremity [9].

**Table 3:** Aerosol-generating procedures in pediatric orthopedic surgery

Surgical procedure	Aerosol-generating level
High speed power tools (saw, burr, drill)	High
Pulsed lavage	High
Electrocautery	High

## Conclusion

The COVID-19 pandemic has dramatically impacted the health systems at a global level. The need for a rapid adaptation and response of the health providers to the pandemic has imposed the suspension of most routine healthcare services, potentially harming children who require COVID19 unrelated care. The SITOP Working Group has drawn up these recommendations with the aim of supporting the decisions of the pediatric orthopaedic surgeons for ensuring continuity of care in children requiring orthopaedic treatments during a pandemic. We are confident that the experience gained across the main Italian pediatric orthopaedic hospitals could be helpful for other professionals involved in children’s care, as well as for pediatric orthopaedic surgeons from other countries, which are facing the pandemic at an earlier phase. The SITOP will also monitor, through multicenter data collection and analysis, the adherence to these recommendations and their safety and effectiveness, to estimate the impact of this coordinated initiative on the health of children with orthopaedic diseases.

## Data Availability

Not applicable.
